# The Warsaw Proposal for the Use of Extended Selection Criteria in Liver Transplantation for Hepatocellular Cancer

**DOI:** 10.1245/s10434-016-5500-0

**Published:** 2016-08-16

**Authors:** Michał Grąt, Karolina M. Wronka, Jan Stypułkowski, Emil Bik, Maciej Krasnodębski, Łukasz Masior, Zbigniew Lewandowski, Karolina Grąt, Waldemar Patkowski, Marek Krawczyk

**Affiliations:** 1 Department of General, Transplant and Liver Surgery, Medical University of Warsaw, Warsaw, Poland; 2 Department of Epidemiology, Medical University of Warsaw, Warsaw, Poland; 3 Second Department of Clinical Radiology, Medical University of Warsaw, Warsaw, Poland

## Abstract

**Background:**

Combination of the University of California, San Francisco (UCSF) and the up-to-7 criteria with alpha-fetoprotein (AFP) cutoff of 100 ng/ml was proposed as the Warsaw expansion of the Milan criteria in selection of hepatocellular cancer (HCC) patients for liver transplantation. The purpose of this retrospective study was to validate this proposal.

**Methods:**

A total of 240 HCC patients after liver transplantation were included. Recurrence-free survival and overall survival at 5 years were set as the primary and secondary outcome measures, respectively.

**Results:**

The Warsaw expansion increased transplant eligibility rate by 20.3 %. AFP >100 ng/ml significantly increased the recurrence risk in patients within the Milan criteria (*p* = 0.025) and in those beyond, yet within either the UCSF or the up-to-7 criteria (*p* < 0.001). Recurrence-free survival at 5 years was 90.8 % for patients within the Milan criteria, 100.0 % in patients within the Warsaw expansion, 54.9 % in patients beyond the Warsaw expansion but within either the UCSF or the up-to-7 criteria, and 45.1 % in patients beyond both the UCSF and the up-to-7 criteria (*p* < 0.001). The corresponding overall survival rates were 71.6, 82.4, 64.3, and 55.3 %, respectively (*p* = 0.027).

**Conclusions:**

The Warsaw expansion of the Milan criteria substantially increases the recipient pool without compromising outcomes.

Liver transplantation is the optimal treatment for selected patients with hepatocellular cancer (HCC) and liver cirrhosis.^1^,^2^ Despite available evidence for the superiority of long-term outcomes of patients undergoing liver transplantation as a first-line management over those undergoing liver resection or salvage liver transplantation, its use remains limited by the worldwide shortage of donors.^3^–^8^ Accordingly, the restrictive Milan criteria (1 tumor <5 cm or 2–3 tumors <3 cm each combined with the absence of extrahepatic lesions or macrovascular invasion) introduced two decades ago are still the gold standard in selecting patients eligible for transplantation, despite the increasing number of reports on the noninferiority of their modest expansion.^9^–^13^ Of numerous morphology-based expansion proposals, the University of California, San Francisco criteria (1 tumor <6.5 cm or 2–3 tumors <4.5 cm with total tumor diameter <8 cm) and the up-to-7 criteria (sum of number of tumors and size of the largest tumor in cm not exceeding 7) appear most popular and widely evaluated.^14^,^15^ However, the risk of posttransplant recurrence is dependent upon morphological tumor burden and long-term survival of patients undergoing liver transplantation for HCC is inferior to those with benign indications even with the widespread use of Milan criteria.^16^–^18^ Therefore, expansion of limits for tumor size and number is subject to criticism.^19^–^21^

Considering the increasing evidence for the independent role of serum tumor markers, particularly alpha-fetoprotein (AFP), in prediction of posttransplant tumor recurrence, modification of the morphology-based criteria using these markers of biological aggressiveness seems not only justified, but also necessary.^22^–^25^ Notably, while serum tumor markers already have been included in several criteria proposals utilized in Asian countries, utilization of such strategy is uncommon in European and American countries, besides a recent French AFP model by Duvoux et al. and the AFP total tumor volume (TTV) criteria by Toso et al.^26^–^32^ In view of geographical discrepancies in the selection criteria and following a detailed set of analyses of the associations between pretransplant AFP and the risk of posttransplant tumor recurrence, AFP was included in the Warsaw proposal for safe extension of Milan criteria. Briefly, it defines patient eligibility either (1) by fulfillment of the Milan criteria, irrespective of pretransplant AFP, or (2) fulfillment of the up-to-7 or UCSF criteria and AFP <100 ng/ml.^33^ In the initial study, the latter subgroup was characterized by the ideal 5-year, recurrence-free survival of 100 %. This proposal was started to be taken under consideration during selection of HCC patients for liver transplantation since the beginning of 2013. Therefore, the purpose of the present study was to evaluate the efficacy of the Warsaw proposal for selection of HCC patients for liver transplantation in provision of acceptable low-risk of post-transplant recurrence.

## Methods

A total of 1602 liver transplantations were performed in the Department of General, Transplant and Liver Surgery at the Medical University of Warsaw (Poland) during the period between December 1989 and April 2015. This retrospective cohort study was performed using the data of 240 patients after liver transplantation for HCC between January 2001 and April 2015. The study protocol was approved by the local ethics committee of the Medical University of Warsaw.

Tumor recurrence at 5-year, follow-up period was set as a primary endpoint of the study, with 5-year recurrence free survival (RFS) being the primary outcome measure. The 5-year RFS was calculated as the time from liver transplantation until diagnosis of recurrence and was censored at the date of last follow-up or death from a nonmalignant cause. The secondary endpoint was patient death at 5 years (irrespective of the cause), which was used to calculate overall survival (OS, secondary outcome measure). Details on the center’s experience, perioperative management, operative technique, immunosuppression protocol, and long-term follow-up were provided elsewhere.^34^,^35^

Primary and secondary outcome measures were evaluated separately in patients (1) within the Milan criteria, (2) beyond the Milan criteria but within the up-to-7 or the UCSF criteria with AFP <100 ng/ml (Warsaw proposal for extension of the Milan criteria), (3) >100 ng/ml, and (4) in patients beyond both the UCSF and the up-to-7 criteria. The particular selection criteria and last pretransplant AFP, as primary variables of interest, were assessed as risk factors for tumor recurrence at 5 years. This was done both in a series of univariable analyses and in multivariable analyses, following adjustment for the impact of other risk factors for rumor recurrence.

Quantitative variables were presented as medians (interquartile ranges) and qualitative variables were presented as numbers (percentages). Kruskal–Wallis test and Chi-square test were used for intergroup comparisons, as appropriate. Kaplan-Meier estimator was used to calculate survival curves and log-rank test was used for the corresponding comparisons. Reverse Kaplan–Meier method was used to calculate median follow-up. Cox proportional hazards regression models were used to evaluate the risk factors for tumor recurrence. Due to the number of events, a series of three variable models were used instead of a one multivariable model. Hazard ratios (HRs) were presented with 95 % confidence intervals (95% CI). The level of statistical significance was set to 0.05. STATISTICA version 12 software (StatSoft Inc., Tulsa, OK) was applied for computing statistical analyses.

## Results

Of 240 patients included in the study, 143 (59.6 %) were within the Milan criteria, 171 (71.3 %) were within the UCSF criteria, and 181 (75.4 %) were within the up-to-7 criteria. Compared with the fulfillment of Milan criteria, utilization of Warsaw extension results in 20.3 % increase in the number of potential HCC recipients (143–172). Comparisons of baseline characteristics between patients within the Milan criteria, beyond the Milan criteria but within the UCSF or the up-to-7 criteria with AFP <100 ng/ml (Warsaw extension), and beyond that limits are presented in Table 1. Besides fulfillment of the particular selection criteria, significant differences between these subgroups were found with respect to AFP concentration (*p* < 0.001), number of tumors (*p* < 0.001), size of the largest tumor (*p* < 0.001), total tumor volume (*p* < 0.001), microvascular invasion (*p* < 0.001), tumor differentiation (*p* = 0.037), and neoadjuvant treatment (*p* = 0.040). Median follow-up was 34 months. In the entire study cohort, 31 patients developed tumor recurrence within 5 years posttransplantation with the RFS rates of 92.3 % at 1 year, 84.8 % at 3 years, and 79.5 % at 5 years. There were 49 deaths in the 5-year posttransplant period with the corresponding OS rates of 89.0, 79.8, and 68.8 %, respectively.

**Table 1:** Comparison of baseline characteristics between patients within the Milan criteria, patients beyond Milan criteria but within the UCSF or up-to-7 criteria with AFP <100 ng/ml (the Warsaw extension), and patients beyond the Warsaw extension

Characteristics	Within Milan criteria (*n* = 143)	Warsaw extension (*n* = 29)	Beyond Warsaw extension (*n* = 68)	*P*
Recipient gender				0.251
Male	100 (69.9 %)	19 (65.5 %)	54 (79.4 %)	
Female	43 (30.1 %)	10 (34.5 %)	14 (20.6 %)	
Recipient age (yr)	57 (52–61)	58 (53–61)	56 (52–61)	0.573
MELD	11 (9–13)	9 (7–12)	11 (8–14)	0.183
HCV infection	100 (69.9 %)	20 (69.0 %)	49 (72.1 %)	0.935
HBV infection	64 (44.8 %)	12 (41.4 %)	26 (38.2 %)	0.664
Within UCSF criteria	143 (100.0 %)	19 (65.5 %)	9 (13.2%)	<0.001
Within up-to-7 criteria	143 (100.0 %)	27 (93.1 %)	11 (16.2 %)	<0.001
Number of tumors	1 (1–2)	2 (1–3)	4 (2–7)	<0.001
Size of the largest tumor (mm)	25 (15–33)	40 (33–51)	45 (35–60)	<0.001
Total tumor volume (cm^3^)	8 (2–22)	44 (28–80)	88 (49–131)	<0.001
Pretransplant AFP (ng/ml)	13 (5–58)	11 (6–21)	114 (14–914)	<0.001
Pretransplant AFP				<0.001
>100 ng/ml	28 (20.4 %)	0 (0.0 %)	34 (50.7 %)	
<100 ng/ml	109 (79.6 %)	29 (100.0 %)	33 (49.3 %)	
Poor tumor differentiation	9 (6.3 %)	3 (10.3 %)	12 (17.6 %)	0.037
Microvascular invasion	22 (15.5 %)	11 (39.3 %)	34 (52.3 %)	<0.001
Neoadjuvant treatment	57 (39.9 %)	19 (65.5 %)	30 (44.1 %)	0.040
Total ischemic time (hr)	9.0 (8.0–10.4)	9.8 (8.5–10.5)	9.3 (8.0–10.3)	0.553
Intraoperative PRBC transfusions (units)	3 (0–6)	3 (2–5)	4 (2–6)	0.420
Intraoperative FFP transfusions (units)	6 (4–10)	7 (5–10)	7 (5–10)	0.232

Data are presented as medians (interquartile ranges) or numbers (percentages)

*UCSF* University of California, San Francisco; *AFP* alpha-fetoprotein; *MELD* model for end-stage liver disease; *HCV* hepatitis C virus; *HBV* hepatitis B virus; *PRBC* packed red blood cells; *FFP* fresh frozen plasma

According to the results of univariable analyses, the Milan criteria (*p* < 0.001), the UCSF criteria (*p* < 0.001), the up-to-7 criteria (*p* < 0.001), and the last pretransplant AFP (*p* < 0.001) were significantly associated with the risk of posttransplant tumor recurrence (Table 2). Other risk factors comprised younger recipient age (*p* = 0.034), presence of microvascular invasion (*p* = 0.029), poor tumor differentiation (*p* = 0.013), and morphological tumor features: number of tumors (*p* < 0.001), size of the largest tumor (*p* = 0.001), and total tumor volume (*p* < 0.001). Notably, AFP >100 ng/ml was a significant risk factor for tumor recurrence in patients within the Milan criteria (HR 7.00; 95 % CI 1.28–38.3; *p* = 0.025) and in those beyond the Milan criteria but within either the UCSF or the up-to-7 criteria (100.0 vs. 54.9 %, *p* < 0.001), however not in patients beyond both the UCSF and the up-to-7 criteria (HR 1.73; 95 % CI 0.70–4.29; *p* = 0.236). Furthermore, a series of multivariable (3-factor) analyses revealed that nonfulfillment of each of the analyzed criteria and AFP >100 ng/ml were independent risk factors for worse 5-year RFS (Table 3). As each of the three particular selection criteria are solely defined by morphological tumor features, number of tumors, size of the largest tumor and total tumor volume were not included in the multivariable models.

**Table 2:** Results of univariable analyses of risk factors for 5-year tumor recurrence

Factors	Hazard ratio	95 % confidence interval	*P*
Male recipient gender	0.84	0.39–1.83	0.663
Recipient age	0.97	0.93–0.99	0.034
MELD	0.99	0.92–1.07	0.814
HCV infection	1.00	0.47–2.13	0.998
HBV infection	1.41	0.69–2.85	0.343
Within Milan criteria	0.22	0.10–0.48	<0.001
Within UCSF criteria	0.25	0.12–0.50	<0.001
Within up-to-7 criteria	0.17	0.08–0.36	<0.001
Number of tumors	1.31	1.18–1.44	<0.001
Size of the largest tumor	1.02	1.01–1.04	0.001
Total tumor volume	1.01	1.01–1.02	<0.001
Pre-transplant AFP >100 ng/ml	4.30	2.05–9.00	<0.001
Poor tumor differentiation	2.90	1.25–6.73	0.013
Microvascular invasion	2.23	1.09–4.57	0.029
Neoadjuvant treatment	1.36	0.67–2.76	0.393
Total ischemic time	1.15	0.94–1.39	0.172
Intraoperative PRBC transfusions	1.00	0.93–1.07	0.986
Intraoperative FFP transfusions	0.98	0.90–1.06	0.626

Hazard ratios were given per: 1 year increase for recipient age; 1 point increase for model for end-stage liver disease; 1 tumor more for number of tumors; 1 mm increase for the size of the largest tumor; 10 cm^3^ increase for total tumor volume; 1 log_e_ increase for alpha-fetoprotein; 1 h increase for total ischemic time; and 1 unit increase for packed red blood cells and fresh frozen plasma transfusions

*MELD* model for end-stage liver disease; *HCV* hepatitis C virus; *HBV* hepatitis B virus; *UCSF* University of California, San Francisco; *AFP* alpha-fetoprotein; *PRBC* packed red blood cells; *FFP* fresh frozen plasma

**Table 3:** Multivariable (3-factor) analyses of the associations between fulfillment of particular selection criteria and AFP over 100 ng/ml and the risk of posttransplant tumor recurrence

Factors	The effects of selection criteria fulfillment and AFP on recurrence-free survival adjusted for					
	Microvascular invasion		Poor tumor differentiation		Recipient age	
	HR (95 % CI)	*P*	HR (95 % CI)	*P*	HR (95 % CI)	*P*
Milan criteria and AFP						
Within Milan criteria	0.20 (0.08–0.50)	<0.001	0.19 (0.08–0.48)	<0.001	0.18 (0.07–0.44)	<0.001
AFP >100 ng/ml	3.98 (1.87–8.47)	<0.001	3.56 (1.69–7.52)	<0.001	3.46 (1.62–7.41)	0.001
UCSF criteria and AFP						
Within UCSF criteria	0.26 (0.12–0.57)	<0.001	0.25 (0.11–0.54)	<0.001	0.23 (0.10–0.50)	<.001
AFP >100 ng/ml	3.83 (1.81–8.12)	<0.001	3.31 (1.55–7.08)	0.002	3.38 (1.58–7.22)	.002
Up-to-7 criteria and AFP						
Within up-to-7 criteria	0.19 (0.08–0.42)	<0.001	0.17 (0.08–0.37)	<0.001	0.18 (0.08–0.38)	<.001
AFP >100 ng/ml	3.60 (1.69–7.64)	<0.001	3.18 (1.50–6.75)	0.003	3.27 (1.53–6.99)	.002

*AFP* alpha-fetoprotein; *HR* hazard ratio; *95 % CI* 95 % confidence interval; *UCSF* University of California, San Francisco

**Fig. 1:**
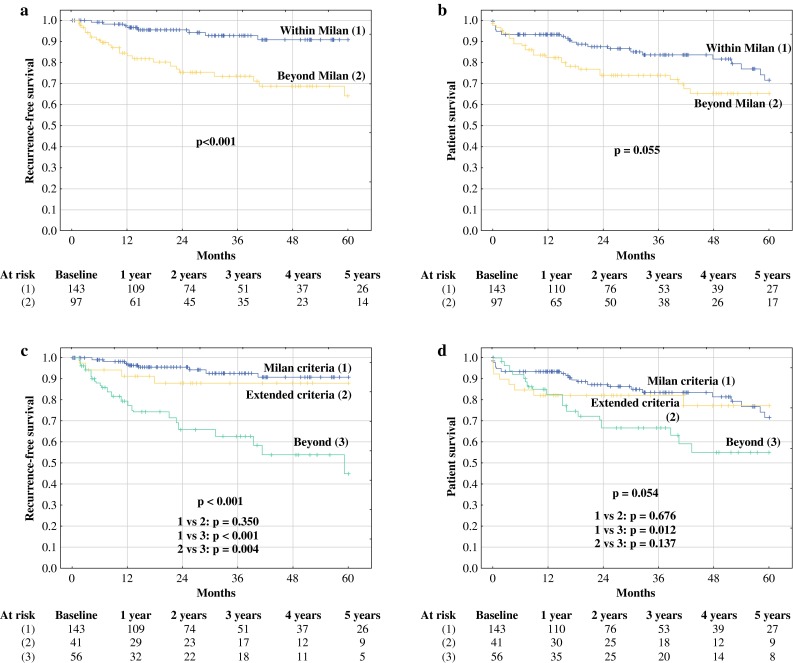
Recurrence-free survival and overall survival curves in patients within (*solid lines*) and beyond (*dashed lines*) the Milan criteria (**a**, **b**) and in patients within the Milan criteria (*solid lines*), beyond the Milan but within either the UCSF or the up-to-7 criteria (*dashed lines*), and beyond (*dotted lines*) both the UCSF and the up-to-7 criteria (**c**, **d**). Numbers of patients at risk are presented below the particular graphs

**Fig. 2:**
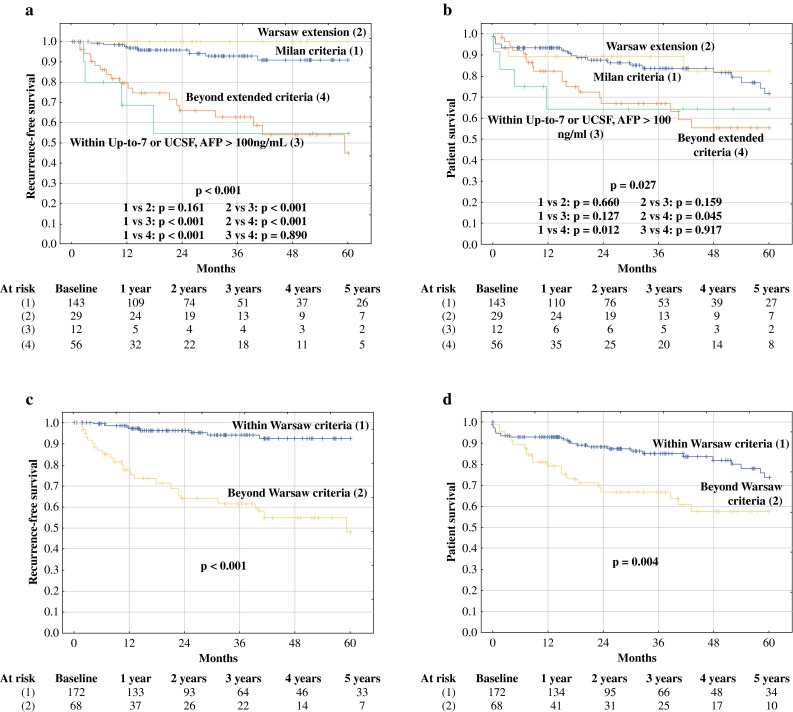
Recurrence-free survival and overall survival curves in patients within the Milan criteria (*solid lines*), beyond the Milan criteria but within the Warsaw expansion (*short-dashed lines*), beyond the Milan criteria and Warsaw expansion but within either the UCSF or the up-to-7 criteria (*dotted lines*), and beyond both the UCSF and the up-to-7 criteria (*long-dashed lines*) (**a**, **b**) and in patients within (*solid lines*) and beyond (*dashed lines*) the Warsaw criteria (**c**, **d**). Numbers of patients at risk are presented below the particular graphs

Patients within the Milan criteria exhibited a 5-year RFS rate of 90.8 %, significantly higher than that observed for patients beyond the Milan criteria (64.2 %, *p* < 0.001; Fig. 1a). The OS rates in the corresponding subgroups were 71.6% and 65.4%, respectively (*p* = 0.055; Fig. 1b). Compared with patients within the Milan criteria, patients beyond Milan but within either the UCSF or the up-to-7 criteria exhibited nonsignificantly lower 5-year RFS of 88.0 % (*p* = 0.350), yet higher than that observed for patients beyond the UCSF and the up-to-7 criteria (45.1 %, *p* = 0.004; Fig. 1c). The only significant difference between these subgroups with respect to OS was found between patients within the Milan criteria and those beyond the up-to-7 and the UCSF criteria (71.6 vs. 55.3 %, *p* = 0.012; Fig. 1d). Notably, RFS at 5 years for patients beyond the Milan criteria but within their Warsaw extension was 100 % compared with 90.8 % in patients within the Milan criteria (*p* = 0.161), 54.9 % in patients beyond the Milan criteria but within either the UCSF or the up-to-7 criteria and AFP >100 ng/ml (*p* < 0.001), and 45.1 % in patients beyond the UCSF and the up-to-7 criteria (*p* < 0.001; Fig. 2a). At 5 years, OS was significantly better for both patients within the Milan criteria (71.6 %) and those beyond the Milan criteria but within their Warsaw extension (82.4 %) compared with patients beyond the UCSF and the up-to-7 criteria (55.3 %, *p* = 0.012, 0.045, respectively; Fig. 2b). Finally, patients within the Warsaw extended criteria exhibited 5-year RFS and OS rates of 92.5 and 73.5%, respectively, compared with the corresponding rates of 47.9 % (*p* < 0.001) and 57.4 % (*p* = 0.004), respectively, in patients beyond these criteria (Fig. 2c, d).

## Discussion

To keep the rates of tumor recurrence after liver transplantation for HCC within the acceptable limits and long-term posttransplant survival of HCC patients comparable to recipients with nonmalignant diseases, the Milan criteria remained the benchmark for assessing patient eligibility for transplantation for the past two decades. The results of previous study from the authors’ department provided the rationale for expansion of these criteria based on the combination of two well-known morphological expansions, namely the UCSF and the up-to-7 criteria, and the biological criterion of AFP <100 ng/ml.^33^ The results of the present study provide further evidence on the lack of any negative effects associated with expansion of the Milan criteria into the Warsaw expanded criteria.

The major disadvantage of the proposals for expansion of Milan criteria based only on morphological features is the potential risk of concomitant increase in the risk of posttransplant tumor recurrence. Despite the optimistic results of prospective validation of the UCSF criteria and the results of several retrospective studies indicating acceptable posttransplant outcomes in patients within either of the two expanded criteria, increase in tumor number and size is a well-known factor associated with the recurrence risk.^15^,^36^–^40^ In fact, the significant effects of tumor number and size have been the basis for creation of the Metroticket model.^15^ Accordingly, patients beyond Milan but within the limits of the UCSF criteria were previously reported to exhibit inferior long-term outcomes.^41^,^42^ In the present study, all morphological tumor characteristics were found to be significantly associated with the risk of posttransplant recurrence. Although the long-term outcomes were not significantly compromised in patients beyond Milan but within either the UCSF or the up-to-7 criteria, this might have been affected by the type II error or, less probably, by the selection bias.

Performed analyses revealed that last pretransplant AFP was an independent risk factor for tumor recurrence; however, the effects varied with respect to fulfillment of different selection criteria. Notably, while the negative impact of AFP >100 ng/ml was statistically and clinically significant in patients within the Milan criteria and beyond the Milan but within the extended criteria, no effects were observed in patients beyond the extended criteria. These results clearly support addition of biological criterion of AFP into the morphological criteria. Most importantly, the null rate of recurrence remained unchanged since the previous analysis, despite both doubling the number of patients and prolongation of the follow-up period for those included in the initial study. Moreover, the findings are further supported by incorporation of the Warsaw extended criteria in the decision-making process regarding selection of patients in the authors’ department since 2013, potentially reducing the risk of selection bias.

Besides several selection criteria utilized in Asia, the concept of combining the biological tumor markers with morphological features is emerging in the Western perspective of liver transplantation for HCC. Important alternatives to the Warsaw extended criteria currently comprise the AFP model introduced by Duvoux et al. and the TTV/AFP introduced by Toso et al.^31^,^32^ Subsequent studies validated the AFP model with respect to both long-term outcomes and net health benefit and the TTV/AFP criteria with respect to long-term outcomes.^43^–^46^ In contrast to these highly relevant proposals of complete redefinition of the Milan criteria, the Warsaw proposal provides an option to expand them in a more conservative fashion, by using the well-established UCSF and up-to-7 criteria. Therefore, AFP cutoff of 100 ng/ml is used in fact as an exclusion criterion, to bring the risk of tumor recurrence associated with increasing tumor burden to or below the level provided by the Milan criteria. Presumably, the use of the Warsaw extended criteria would expand the pool of potential HCC recipients by approximately 20 %, similar to the TTV/AFP criteria and the UCSF criteria, yet lower to the up-to-7 criteria.^47^

Recently, the pretransplant AFP slope has been reported to be a novel predictor of HCC recurrence after liver transplantation.^48^ Nevertheless, this was not confirmed by several other studies, including that of the authors of this manuscript.^49^,^50^ However, the last pretransplant AFP also provides a dynamic assessment of patients eligibility for transplantation, as the AFP values are subject to spontaneous or neoadjuvant treatment-related changes in the pretransplant period.

In conclusion, the results of the present study provide further evidence for the potential lack of negative effects associated with the use of the Warsaw expansion of Milan criteria in selection of patients with HCC for liver transplantation.
